# Supplementary material for: Atrial Fibrillation Related Coronary Embolism: Diagnosis in the Focus
Source: J Pers Med. 2023 Apr 30;13(5):780. doi: 10.3390/jpm13050780 (PMC10221369; doi:10.3390/jpm13050780)
Supplement: Supplementary file 1 [file jpm-13-00780-s001.zip › jpm-2294211-supplementary.pdf]

Supplemental references

1. Acute myocardial infarction due to coronary artery embolism in a patient with atrial fibrillation. Camaro C, Aengevaeren WR.Neth Heart J. 2009 Aug;17(7-8):297-9. doi: 10.1007/BF03086271.PMID: 19789700
2. Atrial fibrillation causing ST elevation myocardial infarction due to coronary embolism: case report and review of the literature. Koutsampasopoulos K, Datsios A, Grigoriadis S, Vogiatzis I.Hippokratia. 2016 Apr-Jun;20(2):160-162.PMID: 28416914
3. Acute Myocardial Infarction Due to Coronary Artery Embolism in a 22-Year-Old Woman with Mitral Stenosis with Atrial Fibrillation Under Warfarinization: Successful Management with Anticoagulation. Sinha SK, Jha MJ, Razi M, Chaturvedi V, Erappa YB, Singh S, Mishra V, Khanra D, Singh K.Am J Case Rep. 2017 Apr 7;18:361-366. doi: 10.12659/ajcr.902250.PMID: 28386054
4. Simultaneously Presented Acute Ischemic Stroke and Non-ST Elevation Myocardial Infarction in a Patient with Paroxysmal Atrial Fibrillation. Kim HL, Seo JB, Chung WY, Zo JH, Kim MA, Kim SH.Korean Circ J. 2013 Nov;43(11):766-9. doi: 10.4070/kcj.2013.43.11.766. Epub 2013 Nov 30.PMID: 24363753
5. Acute Coronary Syndrome (ACS) due to Coronary Artery Embolism in a Patient with Atrial Fibrillation. Daoud H, Abugroun A, Erramilli S, Kumar S.Case Rep Cardiol. 2019 Oct 10;2019:9347198. doi: 10.1155/2019/9347198. eCollection 2019.PMID: 31687218
6. Acute myocardial infarction due to coronary artery embolus associated with atrial fibrillation. Xu B, Williams P, Burns AT.Acute Card Care. 2013 Dec;15(4):92-4. doi: 10.3109/17482941.2013.835828. Epub 2013 Oct 25.PMID: 24160711

7. Acute myocardial infarction due to a coronary embolus during left atrial ablation for persistent atrial fibrillation. Kirubakaran S, Zuberi Z, Gill J.Europace. 2013 Feb;15(2):211. doi: 10.1093/europace/eus194. Epub 2012 Jul 9.PMID: 22778231
8. Coronary embolism causing non-ST elevation myocardial infarction in a patient with paroxysmal atrial fibrillation: treatment with thrombus aspiration catheter. Acikel S, Dogan M, Aksoy MM, Akdemir R.Int J Cardiol. 2011 May 19;149(1):e33-5. doi: 10.1016/j.ijcard.2009.03.077. Epub 2009 Apr 17.PMID: 19375181
9. Acute myocardial infarction and transient ischemic attack in a patient with lone atrial fibrillation and normal coronary arteries. Chlapoutakis GN, Kafkas NV, Katsanos SM, Kiriakou LG, Floros GV, Mpampalis DK.Int J Cardiol. 2010 Feb 18;139(1):e1-4. doi: 10.1016/j.ijcard.2008.06.085. Epub 2008 Aug 19.PMID: 18715661
10. A case of coronary embolism in a patient with paroxysmal atrial fibrillation receiving tamoxifen. Van de Walle S, Dujardin K.Int J Cardiol. 2007 Dec 15;123(1):66-8. doi: 10.1016/j.ijcard.2006.11.091. Epub 2007 Feb 8.PMID: 17291610
11. ST-segment elevation myocardial infarction possibly caused by thromboembolism from left atrial appendage thrombus after incomplete surgical ligation. Guner A, Kılıcgedik A, Kalçık M, Ozkan M.Echocardiography. 2018 Nov;35(11):1889-1892. doi: 10.1111/echo.14123. Epub 2018 Aug 13.PMID: 30105763
12. Acute myocardial infarction secondary to thromboembolism in a patient with atrial fibrillation. Garg RK, Jolly N.Int J Cardiol. 2007 Dec 15;123(1):e18-20. doi: 10.1016/j.ijcard.2006.11.095. Epub 2007 Feb 8.PMID: 17291607
13. Left ventricular mural thrombus and dual coronary embolization associated with hyperthyroid cardiomyopathy

and atrial fibrillation: a case report. Liu G, Yang P, He Y.BMC Cardiovasc Disord. 2017 May 19;17(1):128. doi: 10.1186/s12872-017-0565-7.PMID: 28525971

14. Patient with atrial fibrillation and myocardial infarction due to coronary artery embolism treated with thrombus aspiration. Zasada W, Bartuś S, Królikowski T, Dudek D.Kardiol Pol. 2013;71(1):99-101.PMID: 23348546
15. Cardioembolic acute myocardial infarction and stroke in a patient with persistent atrial fibrillation. Kleczyński P, Dziewierz A, Rakowski T, Rzeszutko L, Sorysz D, Legutko J, Dudek D.Int J Cardiol. 2012 Nov 29;161(3):e46-7. doi: 10.1016/j.ijcard.2012.04.018. Epub 2012 Apr 30.PMID: 22552166
16. Simultaneous cardio-cerebral embolization associated with atrial fibrillation: a case report. Abe S, Tanaka K, Yamagami H, Sonoda K, Hayashi H, Yoneda S, Toyoda K, Koga M.BMC Neurol. 2019 Jul 5;19(1):152. doi: 10.1186/s12883-019-1388-1.PMID: 31277605
17. Paroxysmal atrial fibrillation presenting as anterior wall STEMI in an elderly woman. Shabbir MA, Saad Shaukat MH, Sullenberger L, Torosoff M.BMJ Case Rep. 2019 Sep 4;12(9):e231996. doi: 10.1136/bcr-2019-231996.PMID: 31488453
18. Acute embolic myocardial infarction in a patient with paroxysmal atrial fibrillation receiving direct-current cardioversion. Lin TC, Hsieh YC, Lee WL, Lin YK, Ting CT, Wu TJ.J Chin Med Assoc. 2009 Mar;72(3):146-9. doi: 10.1016/S1726-4901(09)70040-1.PMID: 19299222
19. Coronary Artery Embolism: Two Case Reports and a Review of the Literature. Vendittelli PS, Botros B, Rosman HS, Govindaraju V, Zaitoun A, Marroush TS.Am J Med Sci. 2019 Apr;357(4):333-337. doi: 10.1016/j.amjms.2018.11.003. Epub 2018 Nov 10.PMID: 30545698

20. Incomplete myocardial rupture after coronary embolism of an isolated single coronary artery. Pindado J, Marcos-Alberca P, Rey M, Rábago R, de Diego C, Ibáñez B, Córdoba M, Farré J.Eur J Echocardiogr. 2005 Jan;6(1):72-4. doi: 10.1016/j.euje.2004.07.002.PMID: 15664557
21. Transient ST-segment elevation during transseptal catheterization for atrial fibrillation ablation. Le BH, Black JN, Huang SK.Tex Heart Inst J. 2010;37(6):717-21.PMID: 21224955
22. Coronary embolism causing acute inferior wall and ventricular myocardial infarction in a patient with rheumatic valvular heart disease: treatment with thrombus aspiration. Du XY, Hui P, Zheng Y.Heart Surg Forum. 2015 Jun 26;18(3):E084-7. doi: 10.1532/hsf.1321.PMID: 26115148
23. An uncommon complication of atrial fibrillation. Mallouppas M, Christopoulos C, Watson W, Cader R, Cooper J.Oxf Med Case Reports. 2015 Mar 17;2015(3):232-4. doi: 10.1093/omcr/omv017. eCollection 2015 Mar.PMID: 27559478
24. Cerebral embolism following thrombolytic therapy for acute myocardial infarction: the second reported case. Bostan M, Kanat A, Sen M, Kazdal H, Bostan H.Cardiovasc J Afr. 2010 May-Jun;21(3):155-7.PMID: 20532455
25. Aspiration thrombectomy of a massive thrombotic embolus in acute myocardial infarction caused by coronary embolism. Sakai K, Inoue K, Nobuyoshi M.Int Heart J. 2007 May;48(3):387-92. doi: 10.1536/ihj.48.387.PMID: 17592203
26. Embolic myocardial infarction in atrial fibrillation: multiple territory injury revealed by cardiac magnetic resonance. Dall'Armellina E, Cuculi F, Choudhury RP, Neubauer S, Kharbanda RK.Eur Heart J. 2010 Nov;31(21):2624. doi: 10.1093/eurheartj/ehq232. Epub 2010 Jul 6.PMID: 20605799

27. A Rare Case of Coronary Artery Embolism in a Patient with d-Transposition of the Great Arteries with Prior Mustard Repair. Sethi P, Bhatnagar U, Steffen K, Bendaly E, Stys A.Cureus. 2018 Feb 12;10(2):e2183. doi: 10.7759/cureus.2183.PMID: 29657908
28. Coronary artery saddle embolus and myocardial infarction in a patient with prosthetic mitral valve. Aslam MS, Sanghi V, Hersh S, Lakier JB.Catheter Cardiovasc Interv. 2002 Nov;57(3):367-70. doi: 10.1002/ccd.10316.PMID: 12410516
29. Life-Threatening Acute Occlusion of the Left Main Coronary Artery With Massive Thrombi Originating in the Left Atrial Appendage. Hiraya D, Sato A, Yoshida K, Aonuma K, Ieda M.JACC Cardiovasc Interv. 2018 Sep 24;11(18):e143-e145. doi: 10.1016/j.jcin.2018.07.008. Epub 2018 Aug 29.PMID: 30172798
30. Cardiac arrest due to left circumflex coronary artery embolism as a complication of subtherapeutic oral anticoagulation in a patient with mitral and aortic mechanical valve prostheses. Protasiewicz M, Rojek A, Gajek J, Mysiak A.Postepy Kardiol Interwencyjnej. 2013;9(1):97-100. doi: 10.5114/pwki.2013.34034. Epub 2013 Mar 21.PMID: 24570697
31. Acute myocardial infarction after thrombolytic treatment of acute ischemic stroke. Santos N, Serrão M, Silva B, Pereira A, Faria P, Oliveira R, Caires G, Pereira D, Freitas D, Araújo J.Rev Port Cardiol. 2009 Oct;28(10):1161-6.PMID: 20058780
